# Hemin treatment drives viral reactivation and plasma cell differentiation of EBV latently infected B cells

**DOI:** 10.1371/journal.ppat.1011561

**Published:** 2023-08-28

**Authors:** Anna M. Burnet, Tonya Brunetti, Rosemary Rochford

**Affiliations:** Department of Immunology and Microbiology, University of Colorado, School of Medicine, Aurora, Colorado, United States of America; Brigham and Women’s Hospital, UNITED STATES

## Abstract

Epstein-Barr virus (EBV) and *Plasmodium falciparum* have a well described role in the development of endemic Burkitt lymphoma (BL), yet the mechanisms involved remain unknown. A major hallmark of malarial disease is hemolysis and bystander eryptosis of red blood cells, which causes release of free heme in large quantities into peripheral blood. We hypothesized that heme released during malaria infection drives differentiation of latently infected EBV-positive B cells, resulting in viral reactivation and release of infectious virus. To test this hypothesis, we used the EBV-positive Mutu I B-cell line and treated with hemin (the oxidized form of heme) and evaluated evidence of EBV reactivation. Hemin treatment resulted in the expression of EBV immediate early, early and late lytic gene transcripts. In addition, expression of CD138, a marker of plasma cells was co-expressed with the late lytic protein gp350 on hemin treated Mutu I cells. Finally, DNase-resistant EBV DNA indicative of virion production was detected in supernatant. To assess the transcriptional changes induced by hemin treatment, RNA sequencing was performed on mock- and hemin-treated Mutu I cells, and a shift from mature B cell transcripts to plasma cell transcripts was identified. To identify the mechanism of hemin-induced B cell differentiation, we measured levels of the plasma cell transcriptional repressor, BACH2, that contains specific heme binding sites. Hemin treatment caused significant degradation of BACH2 by 24 hours post-treatment in four BL cell lines (two EBV positive, two EBV negative). Knockdown of BACH2 in Mutu I cells using siRNAs significantly increased CD138+gp350+ cells to levels similar to treatment with hemin. This suggested that hemin induced BACH2 degradation was responsible for plasma cell differentiation and viral reactivation. Together, these data support a model where EBV reactivation can occur during malaria infection via heme modulation, providing a mechanistic link between malaria and EBV.

## Author summary

EBV infection can drive cellular acquisition of cancer hallmarks and is therefore an oncogenic driver of several lymphomas and carcinomas. EBV-associated malignancies require cofactors for initiation and development, and *Plasmodium falciparum* acts as the cofactor for Burkitt lymphoma (BL) pathogenesis. The link between EBV and malaria has long been established, but the exact mechanisms remain unknown. EBV loads are increased in PBMCs and plasma during malaria infection, which begs the question, what about malaria infection can cause reactivation and subsequent expansion of EBV infected cells? From this question, we hypothesized heme released during malaria modulates B cells to drive EBV reactivation and plasma cell differentiation. This hypothesis was tested using hemin, the oxidized form of heme, to treat latently infected B cells at a clinically relevant concentration (pertaining to malaria infection) to measure terminal differentiation and viral reactivation. These results revealed greater understanding of how hemolytic events during malaria impact EBV latency and expand the EBV-positive B cell pool through reactivation and reinfection, therefore increasing the risk of BL.

## Introduction

Infection with *Plasmodium falciparum* causes anemia via hemolysis and bystander eryptosis, which releases large quantities of free heme into the peripheral blood [1–3]. Heme is classified as a damage-associated molecular pattern, or DAMP [4], that acts on immune cells to drive proinflammatory responses, and is associated with cell toxicity and tissue damage [5, 6].

Epstein-Barr virus (EBV) is a human gammaherpesvirus that maintains lifelong latency in B cells. EBV can reactivate from latency following B cell differentiation to plasma cells to produce progeny virus [7]. Plasma cell transcription factors including Interferon regulatory factor 4 (IRF4), X box binding protein 1 (XBP-1), and B lymphocyte-induced maturation protein-1 (BLIMP1, encoded by PRDM1) have been shown to induce EBV lytic replication [8–10]. EBV lytic replication is initiated by two immediate early genes BZLF1 and BRLF1 [7]. This event is followed by early and late viral gene expression, including the late lytic antigen gp350 (encoded by the EBV late lytic gene BLLF1) which is expressed on the cell surface of lytically-infected cells [11].

A high incidence of Burkitt lymphoma (BL) is found where *Plasmodium falciparum* malaria is holoendemic and is a pediatric cancer that is fatal if left untreated [12–15]. Coinfection of EBV and *P*. *falciparum* have been considered causal, yet the mechanisms involved in the pathogenesis of EBV+ BL remain largely unknown. EBV viral loads are increased in PBMCs as well as plasma from children with malaria compared to controls [16,17], and this higher viral load is unmethylated in plasma suggestive that it corresponds to lytic virus [18]. These studies suggest EBV viral reactivation during malaria infection can expand the EBV-positive B cell pool via reactivation and reinfection, setting the stage for increased risk of EBV+ BL [19].

Heme can directly affect B cell transcriptional regulation through interactions with the transcriptional repressor BACH2 via specific binding to five cysteine proline motifs [20]. BACH2 represses plasma cell differentiation [21] through dimerization with Maf proteins to form complexes that blocks the transcription of plasma cell regulators [22,23]. In murine models, heme binding caused degradation of BACH2 [20,24] resulting in differentiation of memory B cells to plasma cells [25]. Additionally, BACH2 is considered a tumor suppressor gene [26,27].

We hypothesized that heme released during malaria infection would drive reactivation and differentiation of latently infected EBV-positive B cells. This hypothesis was tested using BL-derived cell lines to investigate the effect of hemin (the oxidized form of heme) on B cell differentiation and viral reactivation. Our findings have implications for early events in BL pathogenesis where excess heme, a by-product of malaria disease, would induce EBV reactivation from latently infected cells.

## Results

### Hemin induced EBV lytic replication

**Fig 1:**
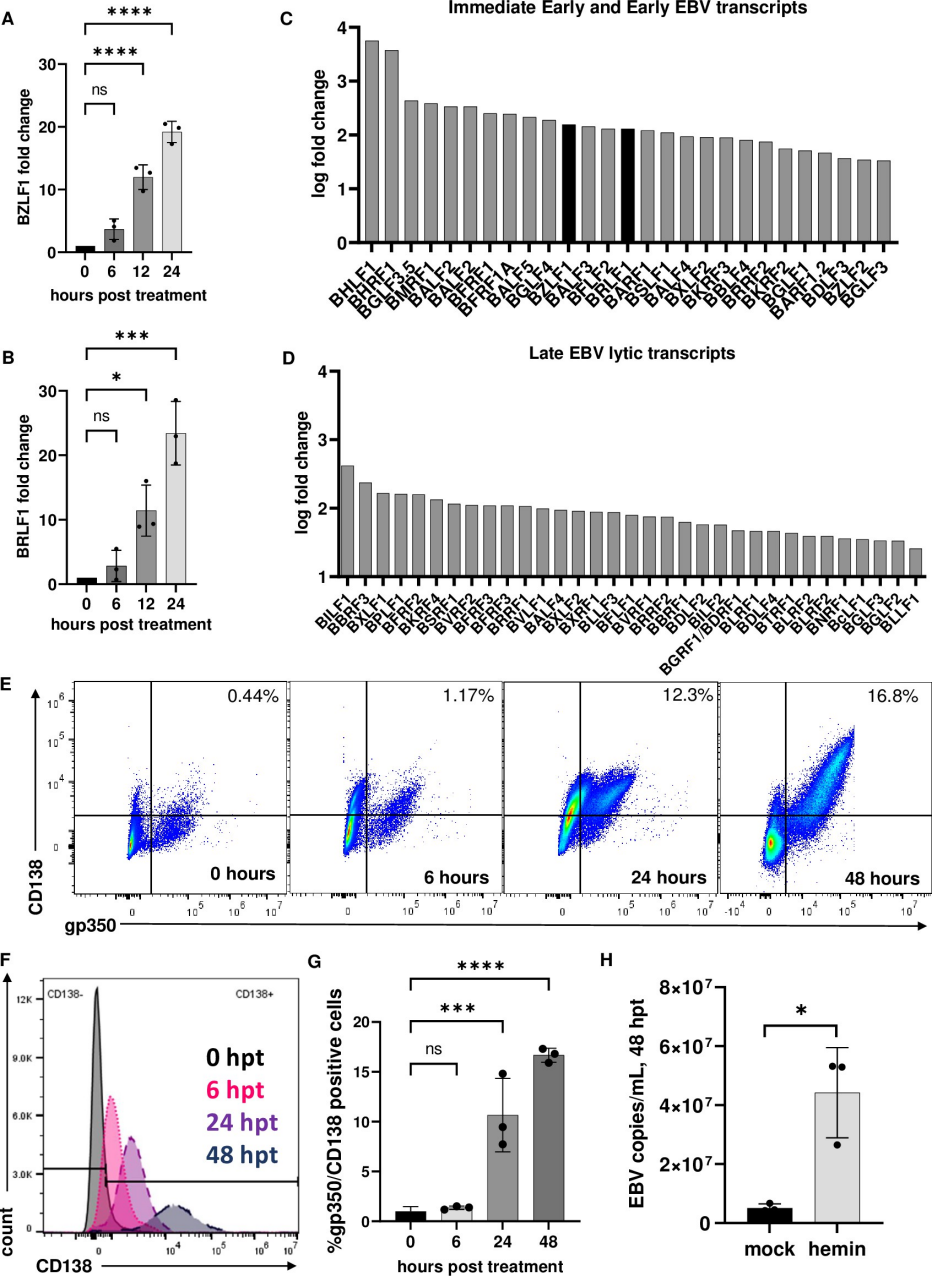
Hemin induced EBV reactivation in Mutu I cells. RNA extracted from Mutu I cells, which exist at ‘latency I’, were analyzed by RT-qPCR for induction of BZLF1 at 0, 6, 12 and 24 hours (A) and BRLF1 (B) demonstrated significant upregulation of both genes at 12 and 24 hours (*p<0.01, ***p<0.0001 ****p<0.0001, n = 3). Immediate early and early significant viral gene log2 transcript fold changes from RNA sequencing (C and D). Flow cytometry analysis of Mutu I cells treated with hemin on a time course of 0, 6, 24 and 48 hours and stained with late lytic antigen gp350 and plasma cell marker CD138 (E, F and G). DNase resistant EBV copies/ mL measured in supernatant from mock and hemin-treated cells, *p<0.01 (H).

The BL cell line, Mutu I, were selected to test our hypothesis as EBV is in a ‘latency I’ status consistent with that state of latency in B cells during viral persistence and BL cell lines have been using to evaluate mechanisms of EBV lytic reactivation [28]. Since EBV reactivation is coupled with plasma cell differentiation [29], we first questioned whether EBV+ Mutu I B cell line treated with hemin could undergo reactivation from latency. To first address this question, a dose response experiment was performed to measure cell viability. Mutu I cells were treated with doses ranging from 62.5 μM to 500 μM. Higher doses of hemin resulted in significant cell toxicity while the lowest dose of 62.5 μM maintained cell viability above 50% for the first three days (S1 Fig). Following this experiment, the concentration of 60 μM was chosen for all subsequent experiments based on a range of free heme concentrations measured in peripheral blood during mild malaria infection and was also the concentration shown to induce primary human memory B cell differentiation *ex vivo* [2,25]. Since 1.4M NaOH was necessary to solubilize hemin, an equivalent amount of NaOH in complete media was used as a ‘mock’ negative control. RNA was extracted from Mutu I cells treated with hemin at 0-, 6-, 12- and 24-hours post-treatment. O-tetradecanoylphorbol-13-acetate (TPA) and sodium butyrate (NaBu), which activate lytic replication and plasma cell differentiation [30,31] served as a positive control for lytic induction (S2 Fig). The expression of the two immediate early (IE) genes, BZLF1 and BRLF1, which are essential for initiation of the EBV lytic cycle was measured by RT-qPCR. Significant induction of both genes was observed by 12 hours and continued through 24 hours post-treatment with a mean fold increase of 19 +/- 1.6 for BZLF1 and a mean fold increase of 22 +/- 4.9 for BRLF1 over mock-treated cells (Fig 1A and 1B, ***p< 0.0003. N = 3).

**Fig 2:**
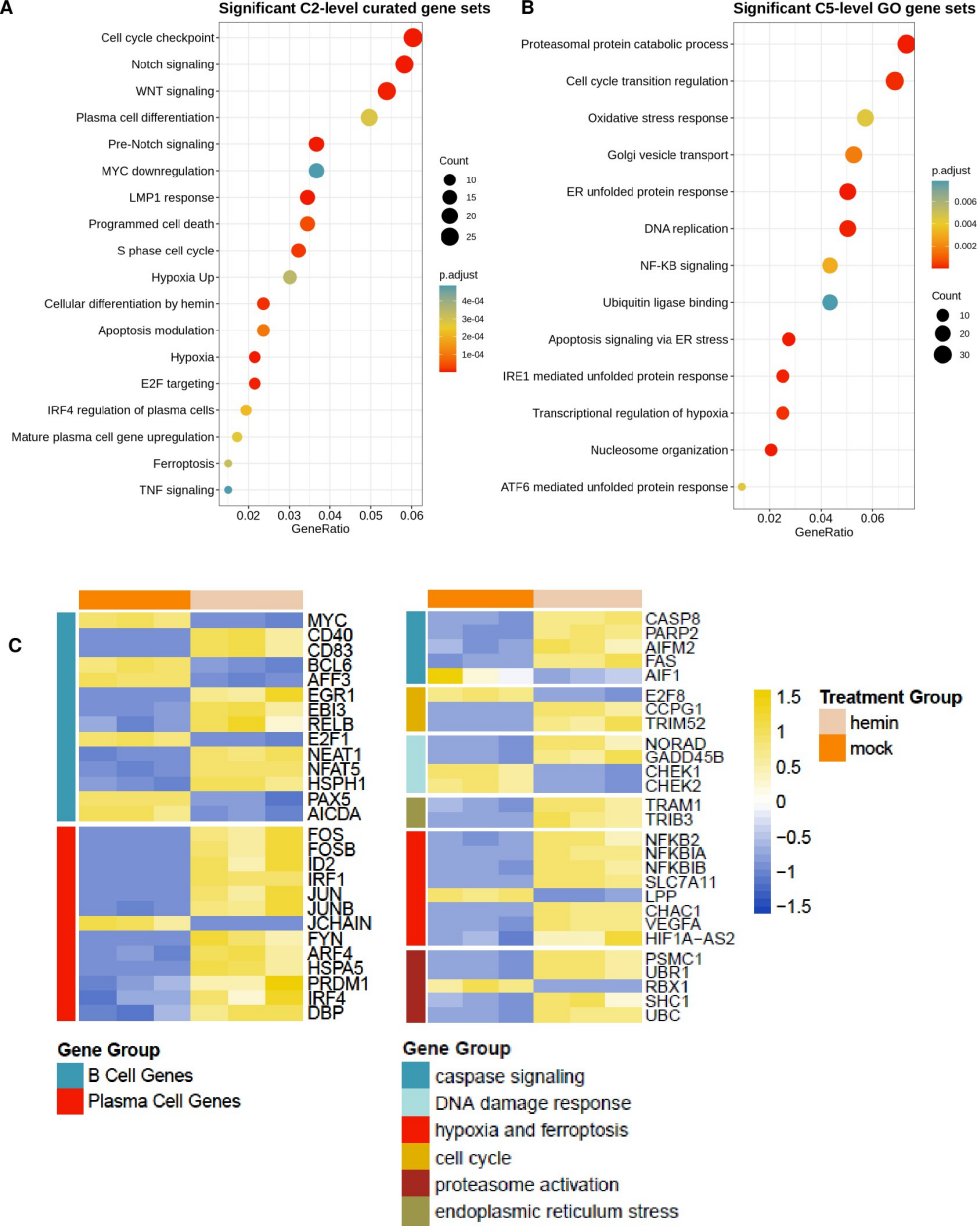
RNA sequencing of mock and hemin treated cells revealed a large transcriptional shift to plasma cell programs. Significant pathway signatures derived from RNA sequencing data illustrated in two gene ontology sets derived from MSigBio, C2 (A) and C5 (B). (C) Heatmap depiction of significant changes in genes related to B cell differentiation and other categories that consist of caspase signaling, hypoxia and DNA damage response. Yellow signifies upregulation while blue represents downregulation. Each column represents experimental replicates (mock/ hemin n = 3). All genes presented in the heat maps were significant and had a false discovery rate (FDR) of less than 1%.

To evaluate whether other EBV lytic transcripts were induced following hemin treatment, we performed RNA sequencing analysis of RNA extracted from Mutu I cells at 24 hours post-mock or hemin treatment). The 24-hour post-treatment timepoint was selected for RNA-seq analysis since this was the height of EBV immediate early gene transcription (Fig 1A and 1B). Total RNA extracted from mock, or hemin treated Mutu I cells (in triplicate) was sequenced at a depth of 60 million reads per sample. Cell viability was measured, with mock averaging 81% +/- 1.5% and hemin averaging 69% +/- 2%, and these viability values for each replicate were used as covariates in a regression analysis to account for viability differences. There was significant upregulation of EBV immediate early, early (Fig 1C) and late lytic genes (Fig 1D). The increased expression of BZLF1 and BRLF1 further confirmed the RT-qPCR results. Further description of the RNA sequencing results on cellular genes is illustrated in Fig 2.

While evidence of increased lytic transcripts was suggestive of lytic reactivation, we next measured whether the late lytic viral glycoprotein gp350 was expressed on the cell surface. To do this, Mutu I cells were treated with hemin over a time course of 0, 6, 24 and 48 hours and analyzed by flow cytometry. In addition, we also evaluated whether CD138, a plasma cell marker, was also expressed (Fig 1F). Flow cytometry analysis of Mutu I at 24 hours post treatment revealed a significant shift to gp350/CD138 dual-positive cells, with further increases in gp350/CD138 dual-positive cells seen at 48 hours post treatment. Significance was determined by a one-way ANOVA with multiple comparisons to zero hours post treatment (Fig 1G).

To confirm that hemin treatment resulted in production and release of EBV virions after lytic replication, cell culture supernatant was collected at 48 hours post hemin treatment based on the timing of lytic transcription and increased dual CD138 and gp350 expression. Supernatant was subjected to DNase treatment, which ensured that only DNA protected in complete virions was measured. Hemin-treated Mutu I supernatant had significant DNase-resistant EBV copy numbers compared to mock supernatant at 48 hours post treatment, confirming there was increased release of virions in following hemin treatment (Fig 1H). Together, this data is consistent with our hypothesis that hemin induced plasma cell differentiation and EBV lytic replication.

### RNA sequencing demonstrated significant pathways in B cell activation and differentiation in hemin treated Mutu I cells

Because we observed EBV lytic transcription and a switch to plasma cells based on CD138 expression, we wanted to measure whether hemin treatment altered the global landscape of transcription in Mutu I cells. A total of 527 genes had significant differential expression (p adjusted value <0.05, false discovery rate of <1%) out of 15,011 total gene hits. A selection of representative genes gathered from curated gene sets illustrated in Fig 2A and 2B are presented in heat maps are shown in Fig 2C. Signatures illustrating plasma cell differentiation, endoplasmic reticulum stress, proteasomal degradation, apoptosis, unfolded protein response, hypoxia, and DNA damage response were commonly found in the significant pathway data sets. The frequency of unfolded protein response and endoplasmic reticulum stress signatures highlight the mechanistic route of plasma cell differentiation further [32–34]. Significant downregulation of important B cell genes were discovered, i.e. *BCL6*, *MYC*, and *PAX5* and significant upregulation of key plasma cell genes, i.e. *PRDM1*, *IRF4*, *FOS* and *JUN*. Other key plasma cell genes such as *XBP-1* were among the hits but remained unchanged.

This large transcriptional shift to plasma cell programs is consistent with hemin treatment inducing terminal differentiation of Mutu I cells. Additionally, this data showed hemin treated Mutu I cells had significant signatures for hypoxia, cellular replication, DNA damage response, oncogenesis and apoptosis, indicating signatures of cellular stress and a broad reorganization of the transcriptional program. These observations were consistent with the danger-molecule nature of hemin, and with viability measurements [4].

### Treatment with hemin drives increased CD138 expression equally between EBV negative and EBV positive cell lines

**Fig 3:**
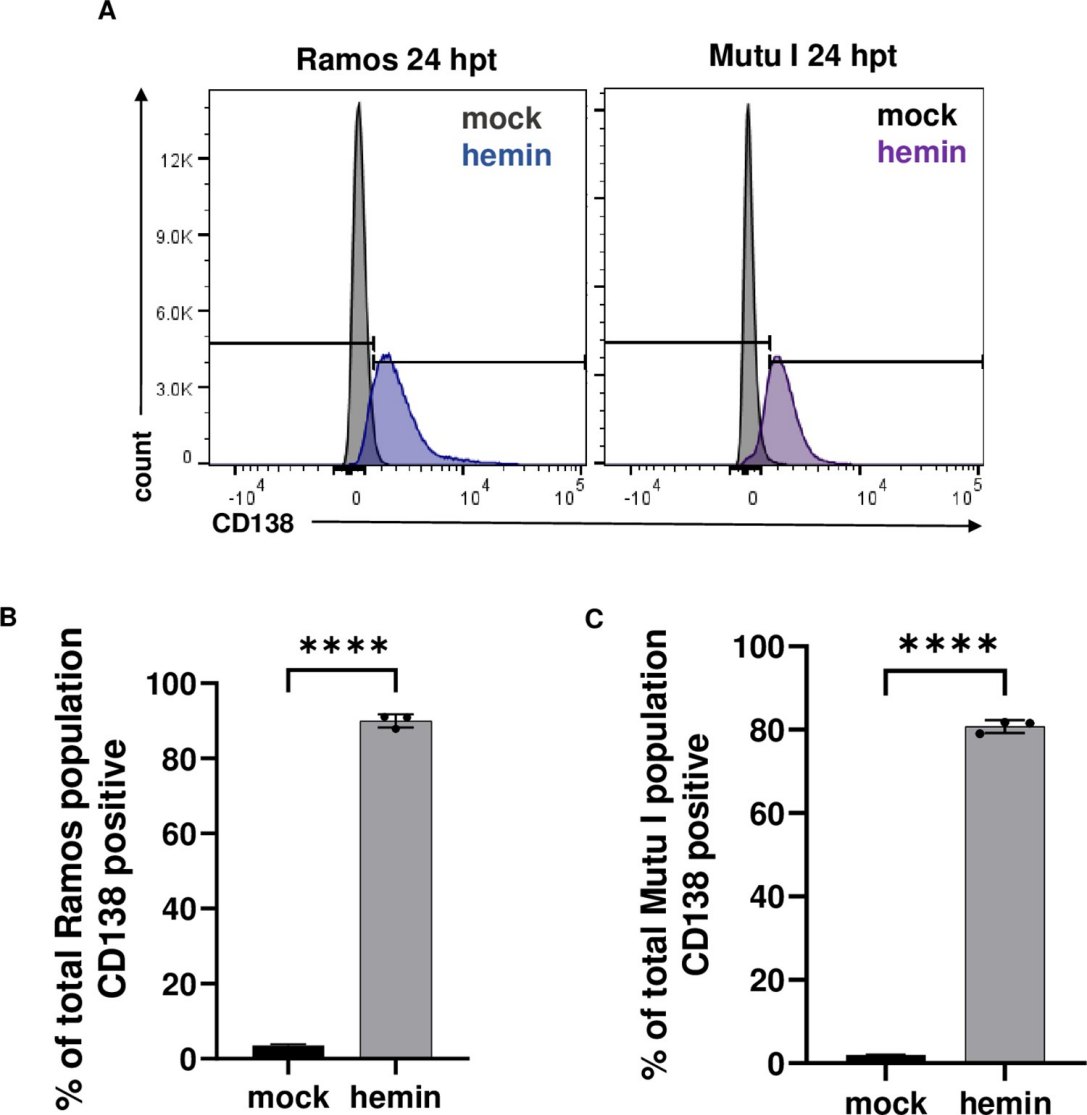
Comparison of plasma cell differentiation following hemin treatment between Ramos (EBV-negative) and Mutu I (EBV positive). (A) Comparison of Ramos and Mutu I CD138 expression at 24 hours post treatment demonstrated significant increases in CD138-positive populations ****p<0.0001 (B and C). N = 3 for all experiments.

Treatment of EBV+ Mutu I cells with hemin resulted in increased CD138 expression on the cell surface and transcriptional changes that indicated the cells had undergone plasma cell differentiation. To determine if increased CD138 expression was specific to an EBV+ B cell line, we compared the effects of hemin treatment for 24 hours on CD138 expression and cell viability (S3 Fig) in both the EBV+ Mutu I BL line and the EBV- Ramos BL line. As shown in Fig 3, hemin treated Ramos and Mutu I cells both had significantly increased CD138- positive populations compared to mock treated cells (Fig 3A and 3B). Ramos had 89% +/- 1.7% CD138-postive cells compared to 3.5% +/- 0.3% in mock, while there were 81% +/- 1.3% CD138-positive cells in hemin treated Mutu I cells compared to 2.8% +/- 0.5% in mock. ****P < 0.0001, N = 3. Differences in cell viability are likely due to cell death via lytic replication.

### BACH2 is degraded by hemin in BL cell lines

**Fig 4:**
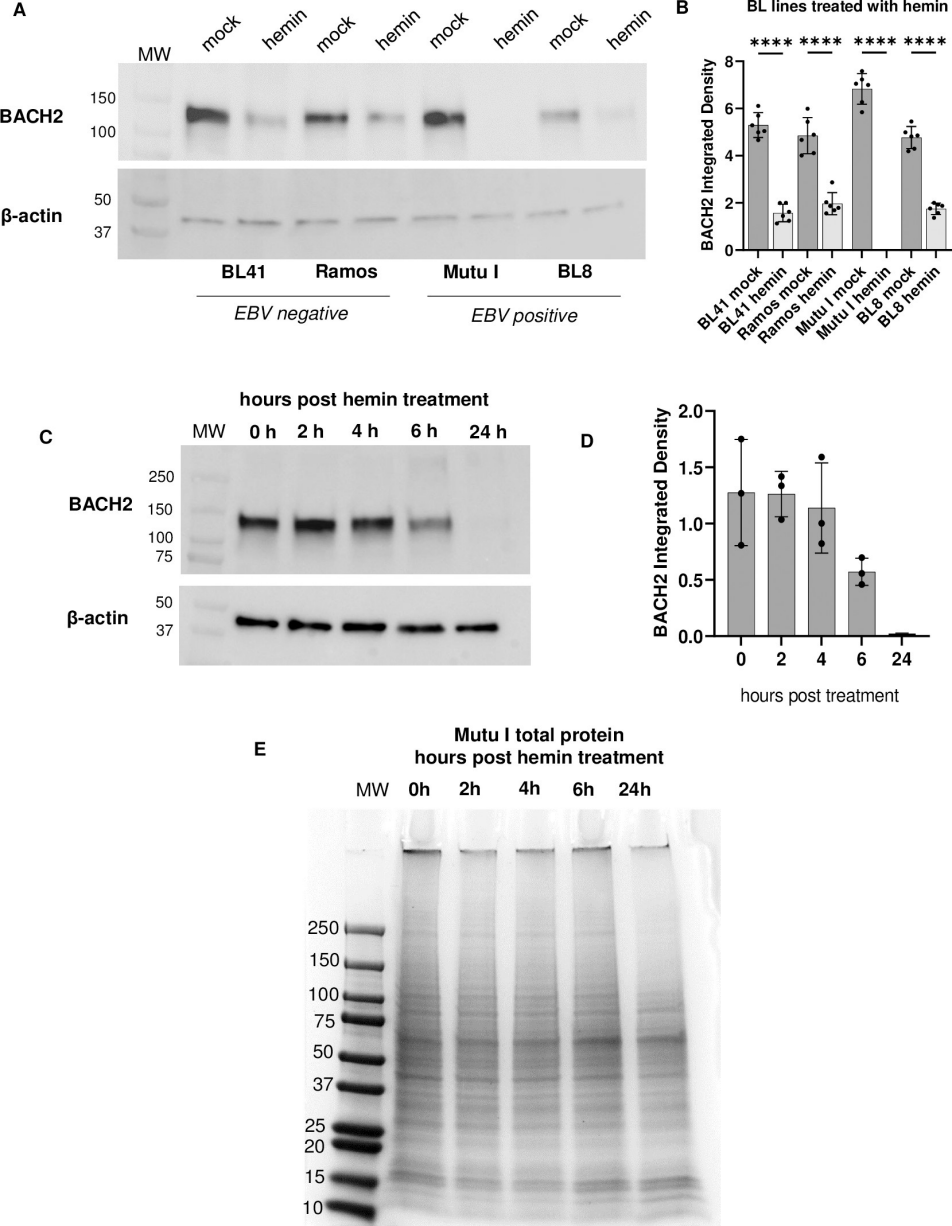
Hemin degraded BACH2 in Burkitt lymphoma cell lines. Protein lysates from EBV-negative (BL41 and Ramos) and EBV-positive (Mutu I and BL8) cell lines were analyzed by western blot for BACH2 protein to measure degradation at 24 hours with Beta actin as a load control (A) and quantified (B). ****p<0.0001 for all cell lines. (C) Time course of BACH2 in Mutu I cells and quantitation in (D). (E) Time course of protein lysates from hemin-treated Mutu I cells, gel stained with Coomassie blue.

To understand how hemin treatment could lead to EBV reactivation, we hypothesized it could be via the release of plasma cell transcriptional repression. Based on previous reports of the targeted effects of hemin on the plasma cell transcriptional regulator, BACH2 in murine B cells [20,24], we tested whether hemin affected BACH2 in BL cells. To investigate this, two EBV negative lines, BL41 and Ramos [30,31] and two EBV positive lines, Mutu I and BL8 [35,36], were evaluated for BACH2 expression following hemin treatment. Protein was extracted at 24 hours post- mock or hemin treatment and BACH2 protein was measured by western blot. Measurement of β-actin was used as a control for equivalent protein loading. All cell lines treated with hemin had significant degradation of BACH2 at 24 hours post-treatment. (Fig 4A and 4B).

To further investigate the effects of hemin treatment on BACH2 degradation, we continued to focus primarily on Mutu I cells. Mutu I cells were treated with hemin for 0, 2, 4, 6 and 24 hours to determine when BACH2 degradation began. Western blot analysis of BACH2 protein indicated that levels of BACH2 began to decline at 6 hours post treatment and was fully degraded by 24 hours (Fig 4C). Quantitation was achieved by measurement BACH2 band density normalized to β-actin band density (n = 3) (Fig 4D).

To determine whether BACH2 degradation by 24 hours post hemin treatment in Mutu I cells was due to cell death and total protein loss, or if BACH2 degradation was a more targeted result of hemin treatment, cell lysates from the same hemin time course experiment shown in Fig 4C were run on a 4–20% gradient gel and stained with Coomassie blue. As seen in Fig 4E, there was no significant decline in total protein through 24 hours post-hemin treatment suggesting that the loss of BACH2 was due to hemin treatment specifically, and not due to non-specific overall protein degradation that would result from cell death.

**Fig 5:**
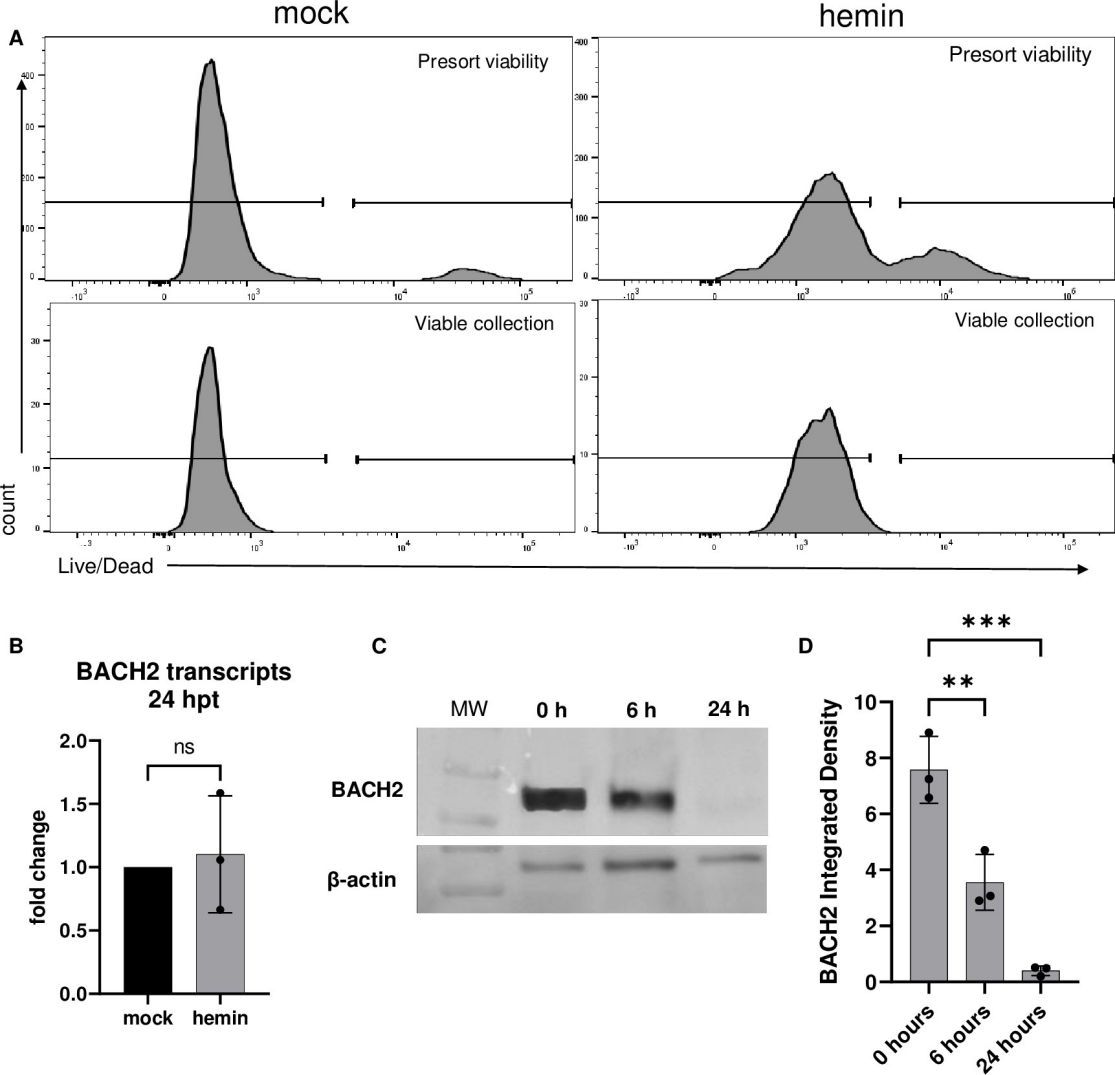
BACH2 is degraded in live cells. Mock or hemin treated Mutu I cells were harvested at 24 hours post treatment and stained with Zombie Aqua viability dye. Cells were sorted on live and dead populations and collected using the gating strategy in A. RNA and protein was extracted from live cells collected by sorting and analyzed by RT-qPCR and western blot. (B) BACH2 transcription 24 hours post treatment. (C) BACH2 protein in live cells sorted at 0-, 6- and 24-hours post hemin treatment, and quantified (D). N = 3.

Because hemin treatment resulted in decreased cell viability (S1 and S3 Figs), we next wanted to verify that BACH2 degradation occurred in live cells. To do this, Mutu I cells were treated with hemin and harvested at 0, 6, and 24 hours and subjected to live/dead cell staining. Cells were then sorted to separate live cells from dead cells using the gating strategy depicted in Fig 5A, and RNA and protein were extracted from the live cells collected. BACH2 transcripts were measured by RT-qPCR from sorted live cells at 24 hours post hemin treatment, which showed BACH2 transcription did not significantly change compared to mock-treated live cells (Fig 5B) (fold change of 1.05 +/- 0.46). This result determined the loss of BACH2 protein at 24 hours post treatment was not due to transcriptional downregulation. Western blot analysis of lysates from live cells demonstrated BACH2 was fully degraded by 24 hours post treatment (Fig 5C) and quantified (Fig 5D, **p<0.002, ***p<0.0001) consistent with the previous results presented in Fig 4A–4C. The significant upregulation of proteasomal degradation signatures in the RNA seq data suggested this is the likely fate of BACH2 following hemin treatment.

To test the stability of BACH2 protein in the presence of hemin, Mutu I cells were treated with cycloheximide (CHX) alone (S5A and S5B Fig) or in combination with hemin (S5C and S5D Fig) to inhibit de novo protein synthesis on a time course of 0, 2, 4, 6 and 24 hours. Protein was extracted and analyzed by western blot. While there was some BACH2 protein loss by 24 hours post CHX alone treatment, when CHX was combined with hemin there was no BACH2 protein by 24 hours post treatment. The half-life of BACH2 is about 5.5 hours [37] and this is further supported by both the observation that BACH2 degradation began at 6 hours post treatment in the hemin time course in Fig 4C, and the decreased BACH2 protein in cycloheximide alone at 6 and 24 hours post treatment in S5 Fig. Based on these observations we concluded BACH2 protein levels were relatively stable (CHX alone) but not in the presence of hemin (S4C and S4D Fig).

*BACH2* is reported to have high rates of mutation in both coding and noncoding regions in BL samples [38–40], so *BACH2* was sequenced in the BL8, Mutu I, BL41 and Ramos cell lines to identify whether there were any mutations relative to the germline sequence. Additionally, we wanted to determine whether any of the five cysteine-proline heme binding sites had mutations that could affect hemin binding. Sequencing of the BACH2 protein coding region in Mutu I, BL8, Ramos and BL41 cells revealed that *BACH2* in these cell lines did not have a high mutation burden, and all cysteine-proline motifs that serve as heme binding sites were unaffected by mutations (S6 and S7 Figs).

### siRNA knockdown of BACH2 increased dual CD138/gp350 expression

Because the effects of hemin treatment on BL cells could be broader than just the degradation of BACH2 protein, we tested whether the selective degradation of BACH2 using siRNAs would also lead to plasma cell differentiation and EBV reactivation. To do this, Mutu I cells were transiently transfected with two BACH2-targeting siRNAs labeled with a TYE transfection control dye, via electroporation at a combined concentration of 250 nM. Transfected cells were analyzed by flow cytometry for CD138 and gp350 expression, and by western blot for BACH2 protein. The 24-hour timepoint was selected foremost for consideration of the responses measured following hemin treatment, but also due to the transient nature of siRNA in these cells and the negative impact of electroporation on cell viability. GAPDH and non-targeting scramble siRNAs served as experimental controls.

**Fig 6:**
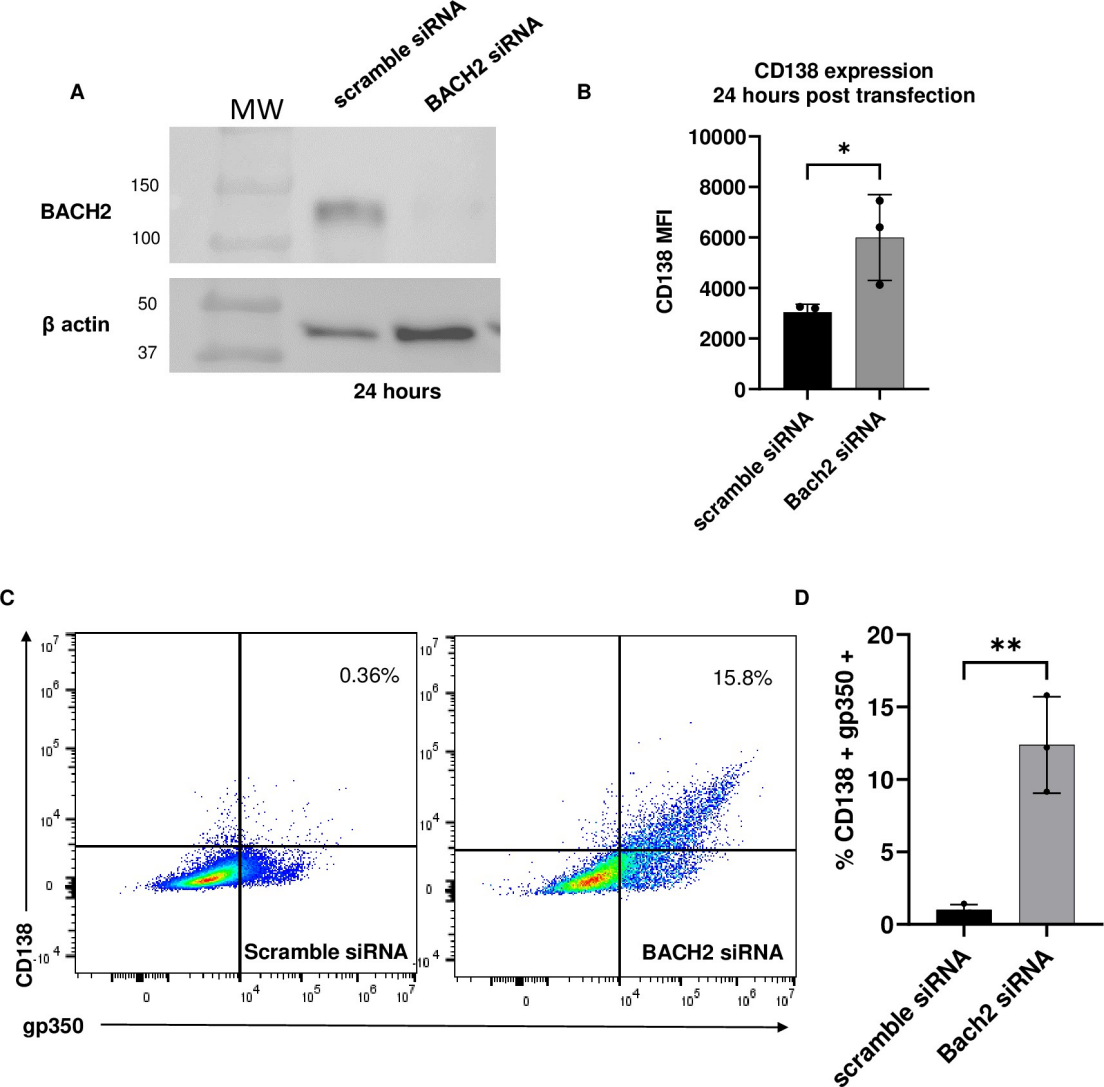
siRNA knockdown of BACH2 increased CD138 and gp350 expression. (A). Western blot analysis of BACH2 protein expression in non-targeting scramble or Bach2 siRNA 24 hours post transfection (B). CD138 MFI in transfected cells was significantly increased in BACH2 knockdown (C). Dual CD138/gp350 positive populations in scramble siRNA compared to Bach2 siRNA were significantly different; **p<0.004 (D). N = 3.

Western blot analysis of protein lysates from scramble or BACH2 targeting siRNA transfected cells demonstrated successful knockdown at BACH2 at the protein level 24 hours post transfection (Fig 6A). Transfection efficiency and cell viability was measured by flow cytometry, and cells were gated on live and transfected populations (S8A Fig). Cells were stained with CD138 at 24 hours post transfection and analyzed by flow cytometry. The BACH2 siRNA treated cells showed significantly increased CD138 MFI compared to non-targeting scramble siRNA treated cells (5995 MFI in BACH2 siRNA compared to 3040 in scramble, *p<0.04 n = 3, Fig 6B), consistent with our model that loss of BACH2 leads to subsequent plasma cell differentiation and lytic reactivation as indicated by increased numbers of gp350+CD138+ cells (Fig 6C and 6D).

BACH2 or scramble siRNA transfections were then stained with CD138 and gp350 at 24 hours for measurement of dual positive populations. A significant difference between experimental groups was observed, about 12% of cells treated with BACH2 siRNA on average were CD138+/gp350+ while less than 0.5% of cells in the scramble siRNA treated cells had dual positive cells (Fig 6C and 6D, **p<0.004. N = 3) which is similar to the level of dual positive cells measured at 24 hours post hemin treatment in Mutu I cells presented in Fig 1F. Collectively, these data show that the consequence of BACH2 degradation was plasma cell differentiation [29] likely through direct interaction of plasma cell elements and viral immediate early gene promoter regions, resulting in viral reactivation [9,10].

## Discussion

EBV reactivation and terminal differentiation of B cells are bound together as synergistic mechanisms. Our studies support a model where hemin induces viral reactivation from latency and plasma cell differentiation via loss of BACH2, a plasma cell transcriptional repressor of BLIMP1, resulting in plasma cell differentiation, directly linking *P*. *falciparum* malaria with EBV reactivation in B cells.

**Fig 7:**
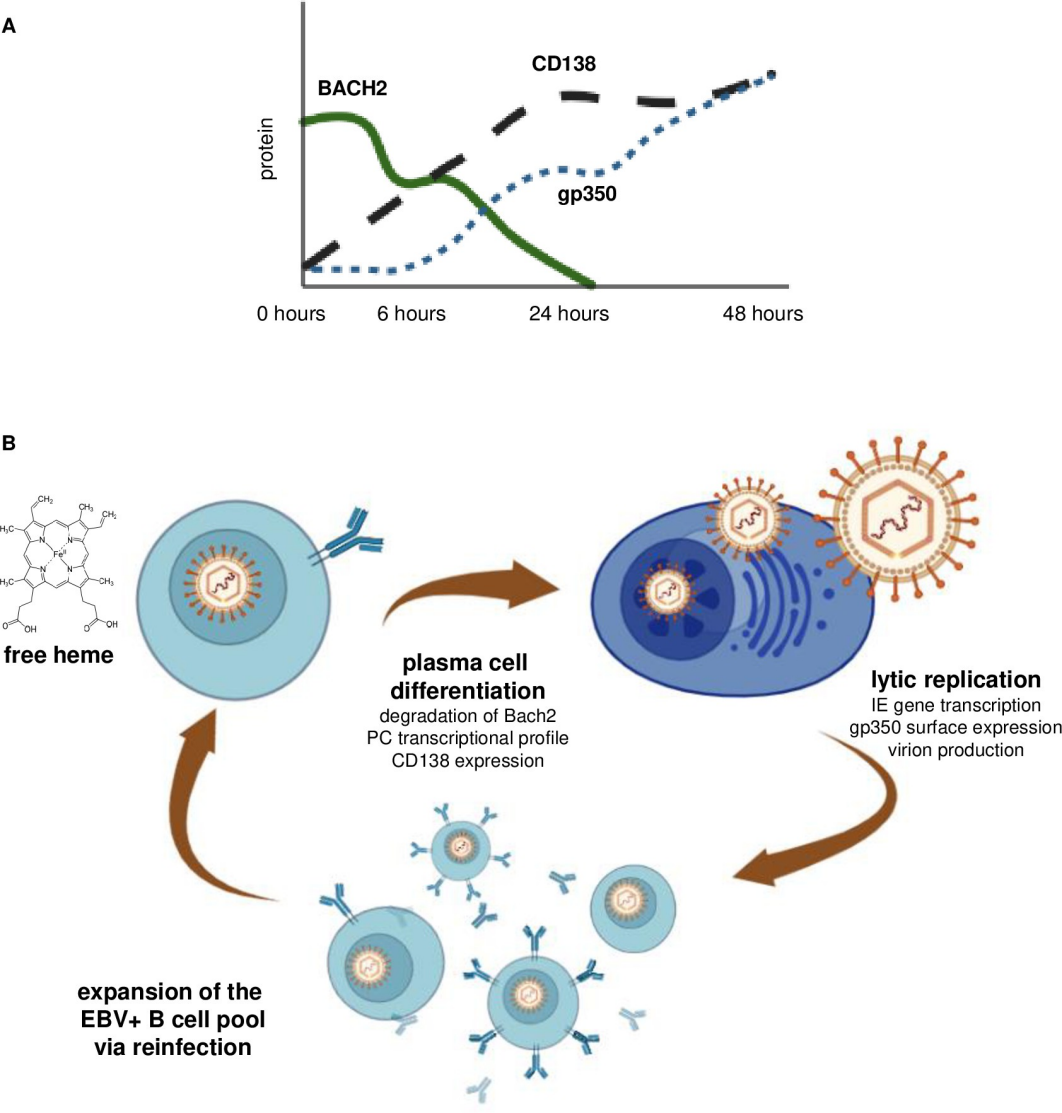
Model of EBV+ B cell pool reinfection via heme modulation. Schematic of BACH2 protein relationship to CD138 and gp350 expression over time (A). Heme drives B cell differentiation to plasma cells, activating EBV lytic replication to produce virions that can infect surrounding cells, ultimately expanding the EBV+ B cell pool to increase the risk for BL (B). Schematics were made in Biorender.

Viral reactivation was verified by immediate early transcript activation, measured by RT-qPCR, further viral lytic transcriptional activity in the RNA seq, expression of late lytic protein gp350 on the surface by flow cytometry that is co-expressed on CD138 positive cells, and detection of DNAse resistant EBV DNA in the supernatant of hemin-treated cells. EBV reactivation can be driven by IRF4, BLIMP1 (encoded by *PRDM1*) and XBP-1 through interaction with immediate early gene promoter elements [8,10,41]. Given the upregulation of both *IRF4* and *PRDM1* in the RNA seq data, both could be responsible for hemin-driven reactivation in Mutu I cells, likely having combinatorial effects either through both interacting with EBV promotors, or through *PRDM1* upregulation by *IRF4*. Additionally, cellular stress signatures in the RNA seq data reflect another effect of hemin, as it is a known damage-associated molecular pattern. Knockdown of BACH2 protein by siRNA recapitulated hemin treatment responses of EBV latently infected B cells, supporting a model of hemin-induced BACH2 degradation, subsequently driving plasma cell differentiation and viral reactivation (Fig 7).

The effects of hemin on B cell differentiation were first reported in mouse model studies [20,24]. More recently, several studies have shown the effects of hemin on primary mouse and human B cells [25,42], Heme has been shown to inhibit plasma cell differentiation in human naïve B cells [42]. In contrast, heme drives plasma cell differentiation in primary human memory B cells, which are epigenetically primed for differentiation to plasma cells and are therefore likely to be more sensitive to heme [25]. EBV persists in memory B cells [43], therefore this B cell subset is of primary interest when considering factors that induce EBV reactivation. In this study, the EBV+ Mutu I BL cell line was used to test the hypothesis that hemin induced EBV reactivation. BL cell lines are thought to derive from late germinal center B cells [44] and express only the EBNA-1 protein, similar to what is found in dividing EBV persistently infected memory B cells in vivo [45] which makes the Mutu I cell line a reasonable model system. An alternative model system to evaluate hemin-induced effects on EBV and B cell differentiation would be the use of EBV-transformed lymphoblastoid cell lines (LCL). The challenge with using LCL to evaluate the effect of hemin are three-fold. First, LCLs express all the EBV latent proteins (latency III) in contrast to the EBNA-1 only expression in latently infected memory B cells. The latency III gene expression program in LCL is more resistant to reactivation signals due to the repressive action of latency proteins on plasma cell genes, namely BLIMP-1 [46]. Second, the LCL-equivalent cell *in vivo* is thought to be found during primary infection of the naïve B cell resulting in a growth phase of the newly infected B cells and thus do not represent the phase of EBV persistence in B cells [47]. Finally, the BACH2 protein is not expressed in LCLs [48]. Because of these limitations, this study focused on hemin-induced effects on BL-derived cell lines. However, a limitation of our study remains that BL cell lines are transformed. The use of non-transformed primary B cells also represents a limitation, as acquiring primary PBMCs from children living in malaria holoendemic regions at cell numbers required for these experiments is not feasible. To our knowledge, there is no *ex vivo* B cell system that expresses EBNA-1 only and isolation and infection of primary B cells with EBV results in expression of all latent genes, making the analysis of hemin effects on BL cells the best available model system.

While heme-driven B-cell terminal differentiation is one route of EBV reactivation, it is not the only mechanism of reactivation that could occur during malaria infections. It would not be surprising that EBV reactivation was multifactorial in malaria endemic regions, as malaria involves a wide range of mechanisms that cause B and T cell dysfunction especially in young children [49–51]. For example, PfEMP1 is a *Plasmodium falciparum* antigen and polyclonal B cell activator that also induces EBV reactivation through its CIDR1α domain [52,53]. Our study is also not the only report of hemolysis and B cell differentiation during malaria: hemolysis-associated phosphatidylserine exposure resulting from malaria infection drives polyclonal plasmablast differentiation [54]. From a different perspective, B cells are activated by TLR ligands, and TLR9 can enhance the efficiency of EBV transformation [55] through downregulation of lytic genes via histone modification. Hemozoin is a malaria parasite byproduct, and a natural ligand of TLR9 [56]. EBV genomes are rich in CpG, which can increase TLR9 expression [57], creating another EBV foothold with the help of malaria. Currently there is no data on whether a relationship between hemin and TLR9 exists, however, hemin can modulate human cells via TLR4 [58], which is expressed in B cells at low levels [59]. Expanding the repertoire of EBV reactivation mechanisms that operate during malaria infection provides more explanation for the high level of detection of EBV infection in children living in malaria endemic regions in Sub Saharan Africa [17,60–63] and ongoing evidence of EBV reactivation [18,64]. Additionally, cellular stress signatures in the RNA seq data reflect another effect of hemin, as it is a known damage-associated molecular pattern [4]. Although our data supports a direct role for hemin induced degradation of BACH2 as a mechanism for EBV reactivation, we cannot rule out the potential effect of cellular stress as a potential inducer of EBV lytic reactivation.

This work has greater implications on how pathogen-driven hemolytic events influence the adaptive immune responses of their hosts via heme modulation of B cells through degradation of BACH2, and even T cells via degradation of BACH1 and BACH2 [65], creating immune dysfunction. Additionally, this may shed light on other poorly understood mechanisms of malaria complications, such as hypergammaglobulinemia [66]. This condition is characterized by an overproduction of antibody, likely driven by hyperactive global plasma cell differentiation, which could be explained by heme modulation of all B cells, regardless of EBV infection.

Hemolytic events and reactivation can be extended beyond malarial disease. Release of heme can also occur on a smaller scale in the oral mucosal compartment at the gumline [67]. This is evidenced by numerous reports on heme-oxygenase 1 induction in the oral cavity, and is of interest in numerous periodontal diseases [68,69]. Since EBV primary infection occurs through the oral route, EBV reactivation and shedding in saliva leading to virus transmission may occur through this mechanism but has yet to be investigated.

The model pathway outlined in Fig 7 illustrates events that occur following bystander eryptosis during malaria infection, which releases free heme in hazardous quantities. This free heme in peripheral blood can be taken up by B cells, either through transferrin receptor binding [70] or heme-carrier protein 1 (HCP1) [71]. Once inside the cell, heme can bind to BACH2 with high specificity at five cysteine-proline motifs, which alters its conformation and causes it to degrade. Degradation of BACH2 results in the release of transcriptional repression of plasma cell elements, allowing cells to terminally differentiate while simultaneously reactivating EBV from latently-infected cells. These reactivation events can cause reinfection of host B cells to increase EBV-positive populations, resulting in augmented EBV loads, and in turn increase the risk for BL.

This study primarily illuminates potential early events in BL pathogenesis that drive EBV reactivation and reinfection of B cells, which leads to increased EBV loads, but it also provides a larger perspective to generate thoughts on the relationship between heme, B cells and how hemolytic events can have far reaching impacts on pathogens and the host environment.

## Materials & methods

### Cell culture

BL41, Ramos, Mutu I and BL8 cells were cultured in ‘complete’ RPMI 1640 medium (Cytiva, Marlborough, MA) containing 10% Equafetal bovine serum (Atlas Biologicals, Fort Collins, CO) 100 μg/mL penicillin-streptomycin and 2 mM glutamax (ThermoFisher, Waltham, MA) at 37°C and 5% CO_2_. Cells were treated withO-tetradecanoylphorbol-13-acetate (TPA) at 25 ng/mL and sodium butyrate (NaBu) at 4 mM, TPA solubilized in EtOH/acetone and NaBu solubilized in H_2_0, or 60 μM hemin (Sigma Aldrich, St. Louis, MO) solubilized in 1.4 M NaOH and compared to mock (NaOH added to complete media only) to measure viral and cellular changes.

### Western blot

Protein lysates were extracted based on cell numbers using RIPA lysis/protein extraction buffer (Cell Signaling Technology, Danvers, MA) with HALT protease cocktail inhibitor (to prevent proteolytic degradation during cell lysis and protein extraction) added fresh. Lysates were quantified using the Pierce BCA Assay (ThermoFisher). Samples were boiled in Laemmli sample buffer (Bio-rad, Hercules, CA) with Beta mercaptoethanol at 70C for 10 minutes prior to being loaded into 4–20% Mini-PROTEAN TGX gels (Bio-rad) and run in a Mini-PROTEAN Tetra Vertical Electrophoresis Cell (Bio-rad) at 100V for 90 minutes. Total protein staining on gels was achieved using GelCode Blue Stain (ThermoFisher) overnight followed by a DI H20 rinse. For westerns, gels were subsequently transferred onto PVDF membranes using the transblot turbo transfer system (Bio-rad) and blocked in 5% non-fat milk for 1 hour. Immunoblot analysis was performed using Rabbit-anti BACH2 at a 1:1000 dilution in (Cell signaling technologies, CA) and stripped and re-probed with β actin (Santa Cruz Biotechnology, Dallas, TX) as a protein load control. Imaging was performed using a Syngene G:Box (Syngene USA, Frederick, MD). Western blot images were quantified in ImageJ (National Institutes of Health) using the integrated density measurement tool. The calculation used to achieve band quantitative values was ((BACH2 value- BACH2 background)/(β actin value-β actin background)).

### RNA sequencing

RNA seq libraries were prepared using paired-end 150 base pair libraries on Mutu I biological replicates. Libraries were prepared, pooled and sequenced at the CU Anschutz Genomics Shared Resource core using the Illumina NovaSeq. All samples were quality screened using FastQC v0.11.9. All reads were trimmed to remove Illumina adapter sequences, and any reads with overall poor quality (q< 24) or too short post-trimming (< 10 base pairs long) were removed using cutadapt v3.4. Samples were aligned to the human genome version GRCh38.p13 using STAR v2.7.3a. Quality of samples and alignment were assessed using Picard Tools v2.21.1 and Samtools v1.8, followed by gene level read quantification using RSEM v1.3.3. All metrics collected from metadata and quality control data were used to identify potential covariates to correct for within the general linear model for differential gene expression analysis using DESeq2. Based on the PCA and linear modeling, cell viability was added as a continuous covariate to our linear model to account for potential differences due to cell death that may affect the differential gene expression analysis. Differential gene expression results were adjusted for multiple comparisons using the Benjamini-Hochberg FDR correction. Any genes with an adjusted p-value < 0.05 and an absolute value of a log2(fold change) >1 were considered differentially significant. All genes considered significant were then used downstream in over-representation pathway analysis. Curated gene sets were obtained from the Molecular Signatures Database (MSigDB), particularly the Hallmark, C2 curated genes and C5 gene ontology genes sets. The R software package, ClusterProfiler, v3.14.3 was used to perform over-representation pathway analysis and to generate the barplot and dotplot gene set enrichment graphs. A representative selection of genes belonging to significant gene sets in Hallmark, C2, and C5 were used to generate the heatmap. The heatmap represents normalized counts extracted from DESeq2 and was generated using the R software package, heatmap v1.0.12.

### BACH2 sequencing

DNA from Mutu I, Ramos, BL41 and BL8 was extracted using the Qiagen DNA extraction kit and subjected to PCR amplification using Phusion Hot Start 2X master mix (New England Biolabs, Ipswich, MA) to capture the BACH2 transcriptional regulator sequence that consists of 5 exons using the following primer sets (Integrated DNA Technologies, Coralville, IA): Exon 4, size 586bp: FWD: 5’- GCTGATTGGTAATTCCCCTCCTTCC-3’, REV: 5’-GTGCCTCTGAGACTTTAAGTGTATC-3’, Exon 5, Size 963bp: FWD: 5’-GTACTACAAGATTAACTGCCCCTTTGC-3’, REV: 5’-CAGGCCAGACAGCTCCACACTTTTC-3’, Exon 6, Size 942bp: FWD: 5’-GCCTGGAGAGATCCAGGAGC-3’, REV: 5’-CAATGTGGGAGTGGTGGG-3’, Exon 7, Size 485bp: FWD: 5’-CATTTTTGATGTTCCGCCTGCC-3’ REV: 5’-CATGCATGCGCGTGCAGACAC-3’, Exon 8: 812bp: FWD: 5’-GTCCTGGGGAAGGTGTTGGC-3’, REV: 5’-GCACCAAATGTGTTCTCGGTTTCTTCG-3’. All PCR products were run on a 2% agarose gel prior to being sent to QuintaraBio (Hayward, CA) for sequencing. Sequencing results were analyzed using Chromas, Sequencher (Gene Codes Corporation, Ann Arbor MI) and the Clustal Omega multiple sequence alignment tool. Sequences from BL lines were compared against two template sequences: NCBI BACH2 sequence downloads and HC04 DNA sequences included in these experimental groups as a control.

### Flow cytometry and cell sorting

Mock, TPA/NaBu and hemin treated Mutu I cells were harvested, live dead stained using Zombie Aqua dye (Biolegend, Burlington, MA) for 30 minutes in PBS followed by blocking for 20 minutes at 4°C (FACS + 5% BSA with Fc block at a 1:100 dilution). Cells were then washed with fluorescence-activated cell sorting (FACS) buffer (phosphate-buffered saline containing 0.5% BSA) and stained with mouse-anti gp350 for 30 minutes at 4°C, washed with FACS buffer and stained with goat anti mouse Alexa Fluor 488 IgG2 secondary (ThermoFisher), and anti-human CD138 Alexa Fluor 647 (Biolegend) antibody for 30 minutes at 4°C, washed with FACS buffer three times and fixed with 1% paraformaldehyde. Cells were analyzed using a Fortessa flow cytometer (BD Biosciences, San Jose, CA) and a Cytek Aurora flow cytometer (Cytek Biosciences, Fremont, CA) 200,000 events (1 event = 1 cell) were collected for all samples. Cells were sorted using a BD Aria I and 800,000 cells were collected per live cell population. All data was analyzed using FlowJo software (Tree Star, Ashland, OR) and GraphPad Prism (San Diego, CA).

### Real time quantitative PCR

RNA was extracted from mock, TPA/NaBu and hemin-treated cells using the Quick RNA MiniPrep Plus kit (Zymo Research, Irvine CA). cDNA synthesis was performed using the Applied Biosystems cDNA reverse transcription kit (Applied Biosystems,

Waltham, MA). Samples were analyzed by RT-qPCR run on the Applied Biosystems Viia7 machine using the following primers: housekeeping gene β-actin- forward primer: 5’–TCACCCACACTGTGCCCATCTACGA– 3’, reverse primer: 5’–CAGCGGAACCGCTCATTGCCAATGG– 3’, probe: 5’–/5HEX/ATGCCCTCCCCCATCCATCCTGC GT/3BHQ_1/– 3’. BZLF1 forward primer 5’–ACGACGCACACGGAAACC– 3’, reverse primer 5’–CTTGGCCCGGCATTTTCT– 3’ and probe 5’–FAM/GCATTCCTCCAGCGATTCTGGCTGTT/BHQ_1–3’. BRLF1 forward primer: ACCATACAGGACACAACACTTC, reverse primer: 5’-GATGTTGAGCGTGGCCATTAGC– 3’ and probe: 5’-FAM/GTTAGCCTCAGAAAGTCTTCCAAGCCATCC– 3’ (IDT Technologies, Coralville, IA). Heme Oxygenase-1 (HO-1) forward primer: 5’-GCAGAGAATGCTGAGTTCATG-3’ reverse primer: 5’- CACATCTATGTGGCCCTGGAGGAGG-3’. Cycling conditions were 50°C hold for 2 minutes, followed by 40 cycles of 95°C for 30 seconds, 60°C for 1 minute. Results were analyzed by the ∆ cycle threshold, ∆∆Ct method. For virion quantification, DNA was extracted using the Qiagen DNA mini kit (Qiagen, Germany) and analyzed by qPCR using β-actin and EBV BALF5, forward primer 5’–CGGAAGCCCTCTGGACTTC– 3’, reverse primer 5’–CCCTGTTTATCCGATGGAATG– 3’ and probe: 5’—/56-FAM/TGTACACGCACGAGAAATGCG CCT/3BHQ_1/ - 3’.

### siRNA knockdown

Mutu I cells were plated in opti-MEM (ThermoFisher) for 18 hours, then resuspended in Mirus Ingenio buffer (Mirus Bio, Madison, WI) that contained siRNAs targeting GAPDH, BACH2, or scramble at a concentration of 250 nanomolar, and transferred to 0.2 cm Ingenio cuvettes (Mirus Bio). Two 27mer ‘dicer’ siRNAs were used to target BACH2 at a combined concentration of 250 nanomolar (IDT technologies). Transfections via electroporation were achieved using the Amaxa II nucleofector system (Lonza, Basel, Switzerland), specifically the B cell protocol A-024. Immediately after electroporation, cells were placed in medium and harvested 24 hours later for RNA, protein lysates and live cells for flow cytometry analysis.

### Statistical analysis and data availability

Statistical analysis was performed using GraphPad Prism. All experiments were run at least in triplicate. Statistical significance for western blots, RT-qPCR, and flow cytometry results were determined using a one-way ANOVA with multiple comparisons when including TPA and NaBu controls, and a student’s T test for comparing only mock and hemin or scramble and BACH2 siRNA. The data that support the findings of this study are publicly available from Dryad repository: Burnet, Anna (2023), Data from: Hemin treatment drives viral reactivation and plasma cell differentiation of EBV latently infected B cells, Dryad, Dataset, https://doi.org/10.5061/dryad.cnp5hqc9j.

### Dryad DOI

https://doi.org/10.5061/dryad.cnp5hqc9j [72]

## Supporting information

S1 Fig Hemin dose titration and cell viability. To determine the relationship between hemin dosage and cell viability, Mutu I cells were untreated or treated with hemin at concentrations of 62.5, 125, 250 and 500 μM and cell viability was measured every 24 hours for 4 days. The lowest concentration of 62.5 had the highest viability.
(TIF)

S2 Fig TPA/NaBu treatment of Mutu I cells as a positive control. (A) BZLF1 and BRLF1 transcripts measured in TPA/NaBu-treated Mutu I cells by RT-qPCR. (B) Gating strategy for CD138/gp350 staining experiments on Mutu I cells treated with TPA/NaBu. (C) Dual positive CD138/gp350 populations 48 hours post TPA/NaBu treatment. (D) Cell viability measurements following TPA/NaBu treatment.
(TIF)

S3 Fig Comparison of viability post hemin treatment between Ramos and Mutu I. Time course of EBV- negative Ramos (A) cell viability post hemin treatment and EBV positive Mutu I (B) cell viability post hemin treatment.
(TIF)

S4 Fig Gating strategy for Mutu I and Ramos flow cytometry analyses. Mutu I (A) and Ramos (B) BL cell lines were analyzed in FlowJo by gating in the order of lymphocytes, single cells, then live dead.
(TIF)

S5 Fig BACH2 stability using cycloheximide. Stability of BACH2 was measured using cycloheximide (CHX) alone (A) and quantified (B) and in combination with hemin (C) and quantified (D). N = 3 for all western blots.
(TIF)

S6 Fig BACH2 sequencing. (A) Schematic of the BACH2 gene, corresponding mRNA with exons shown in yellow, and the resulting transcriptional regulator protein shown in tan. Dotted lines represent primers used to capture exons that encode for the transcriptional regulator. (B) Schematic of the BACH2 transcriptional regulator with prominent features in green (BTB domain, bZIP- basic leucine zipper, CLS-cytoplasmic localization signal) and the five cysteine-proline motifs that serve as heme binding sites shown in red, labeled with their amino acid positions. (C) PCR products from exons 4 through 8 for each cell line were run on a DNA agarose gel.
(TIF)

S7 Fig BACH2 transcriptional regulator sequences from BL cell lines. BACH2 transcriptional regulator sequences derived from Mutu I, Ramos, BL41, and BL8 and compared to the NCBI BACH2 reference sequence. Cysteine proline motifs that serve as heme binding sites are highlighted in yellow.
(TIF)

S8 Fig Gating strategy for BACH2 siRNA experiments. (A) BACH2 siRNA or scramble cells were gated on lymphocytes, single cells, live cells, then transfected cells. (B) CD138 expression compared between scramble and BACH2 siRNA.
(TIF)
