# Supplementary figures and images for: Hemin treatment drives viral reactivation and plasma cell differentiation of EBV latently infected B cells
Source: PLoS Pathog. 2023 Aug 28;19(8):e1011561. doi: 10.1371/journal.ppat.1011561 (PMC10491393; doi:10.1371/journal.ppat.1011561)

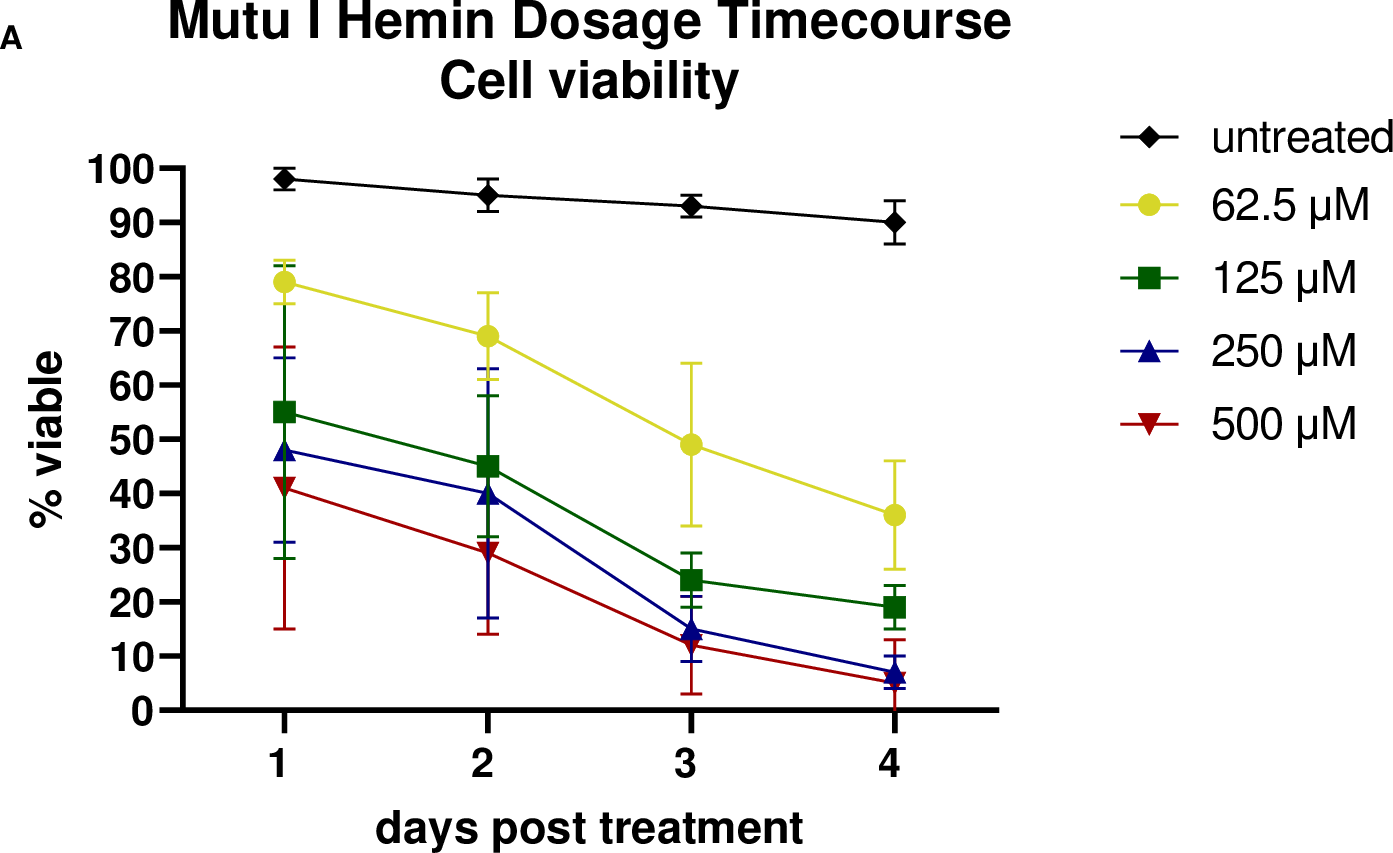

Supplement: S1 Fig — To determine the relationship between hemin dosage and cell viability, Mutu I cells were untreated or treated with hemin at concentrations of 62.5, 125, 250 and 500 μM and cell viability was measured every 24 hours for 4 days. The lowest concentration of 62.5 had the highest viability. (TIF) [file ppat.1011561.s001.tif]

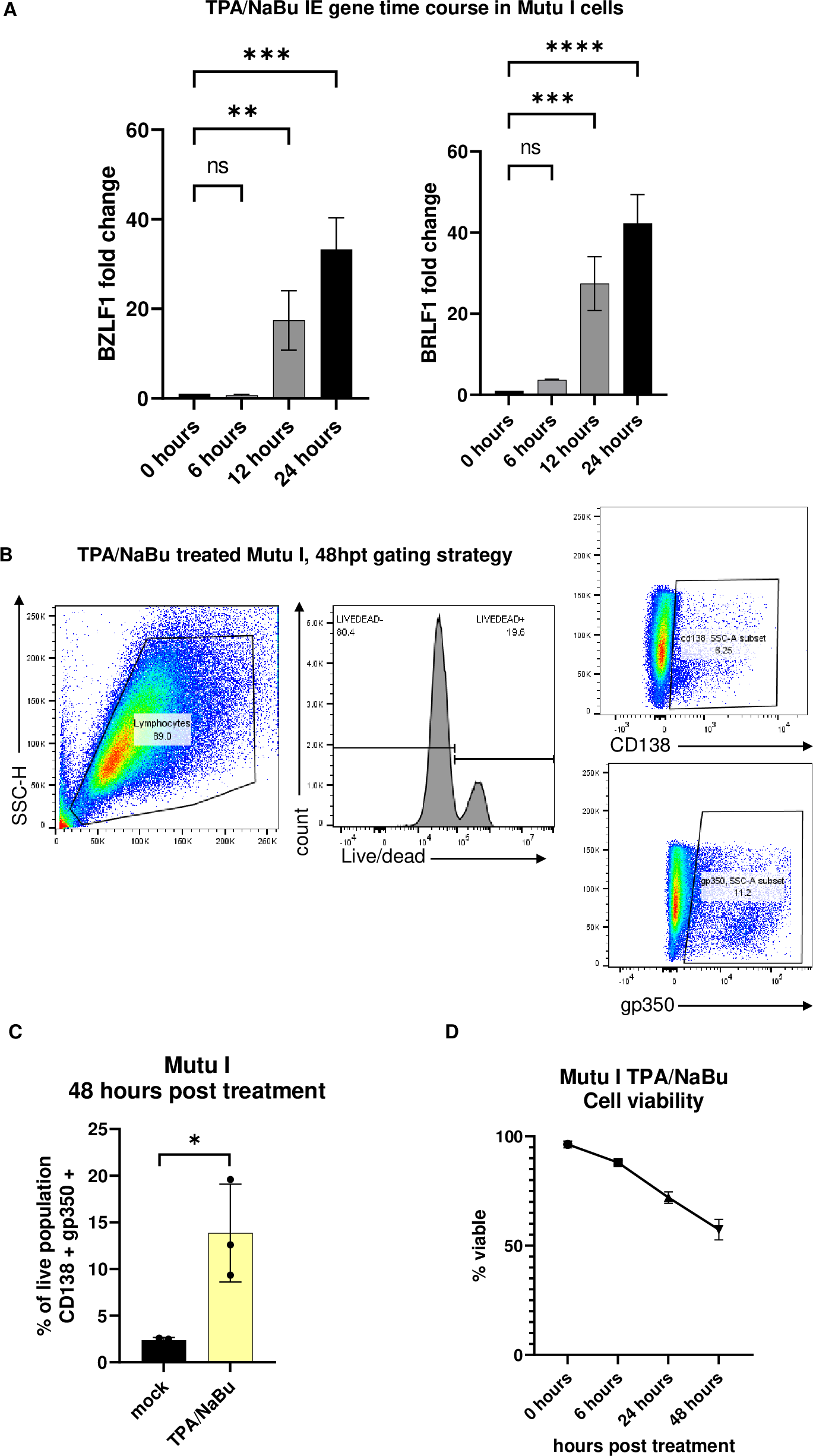

Supplement: S2 Fig — (A) BZLF1 and BRLF1 transcripts measured in TPA/NaBu-treated Mutu I cells by RT-qPCR. (B) Gating strategy for CD138/gp350 staining experiments on Mutu I cells treated with TPA/NaBu. (C) Dual positive CD138/gp350 populations 48 hours post TPA/NaBu treatment. (D) Cell viability measurements following TPA/NaBu treatment. (TIF) [file ppat.1011561.s002.tif]

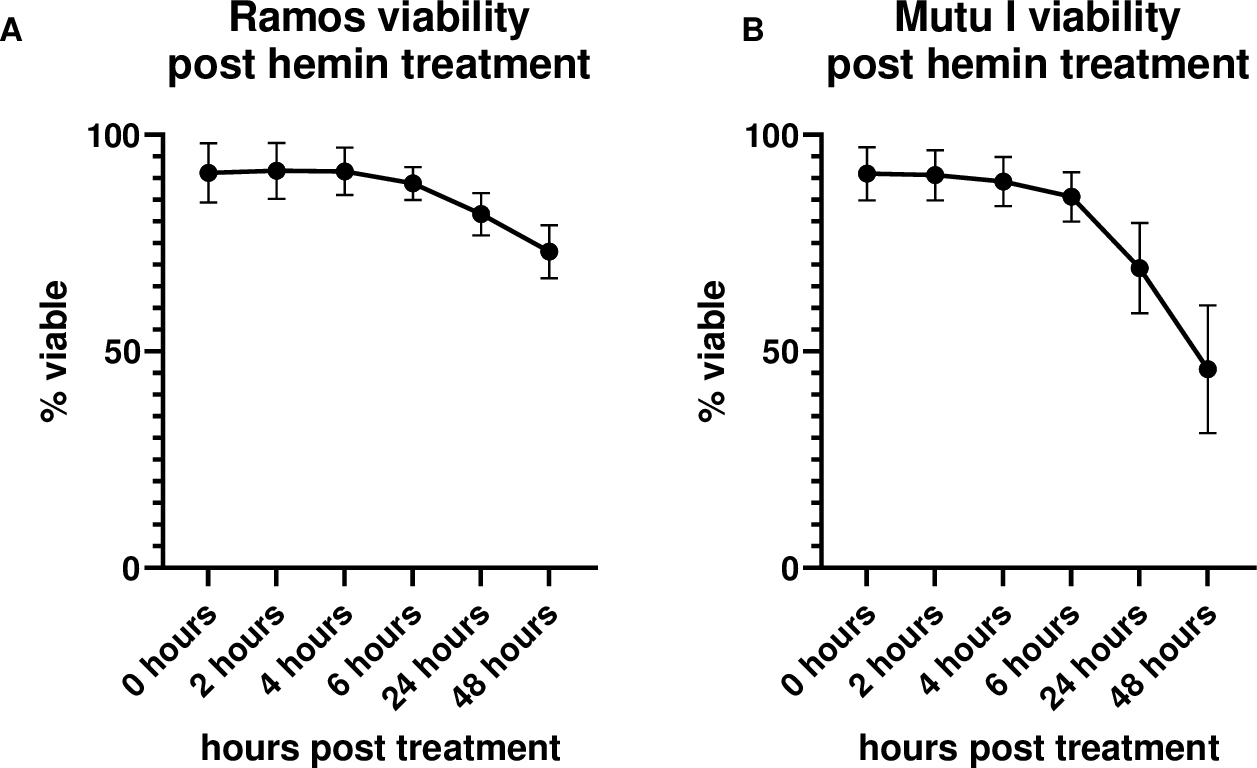

Supplement: S3 Fig — Time course of EBV- negative Ramos (A) cell viability post hemin treatment and EBV positive Mutu I (B) cell viability post hemin treatment. (TIF) [file ppat.1011561.s003.tif]

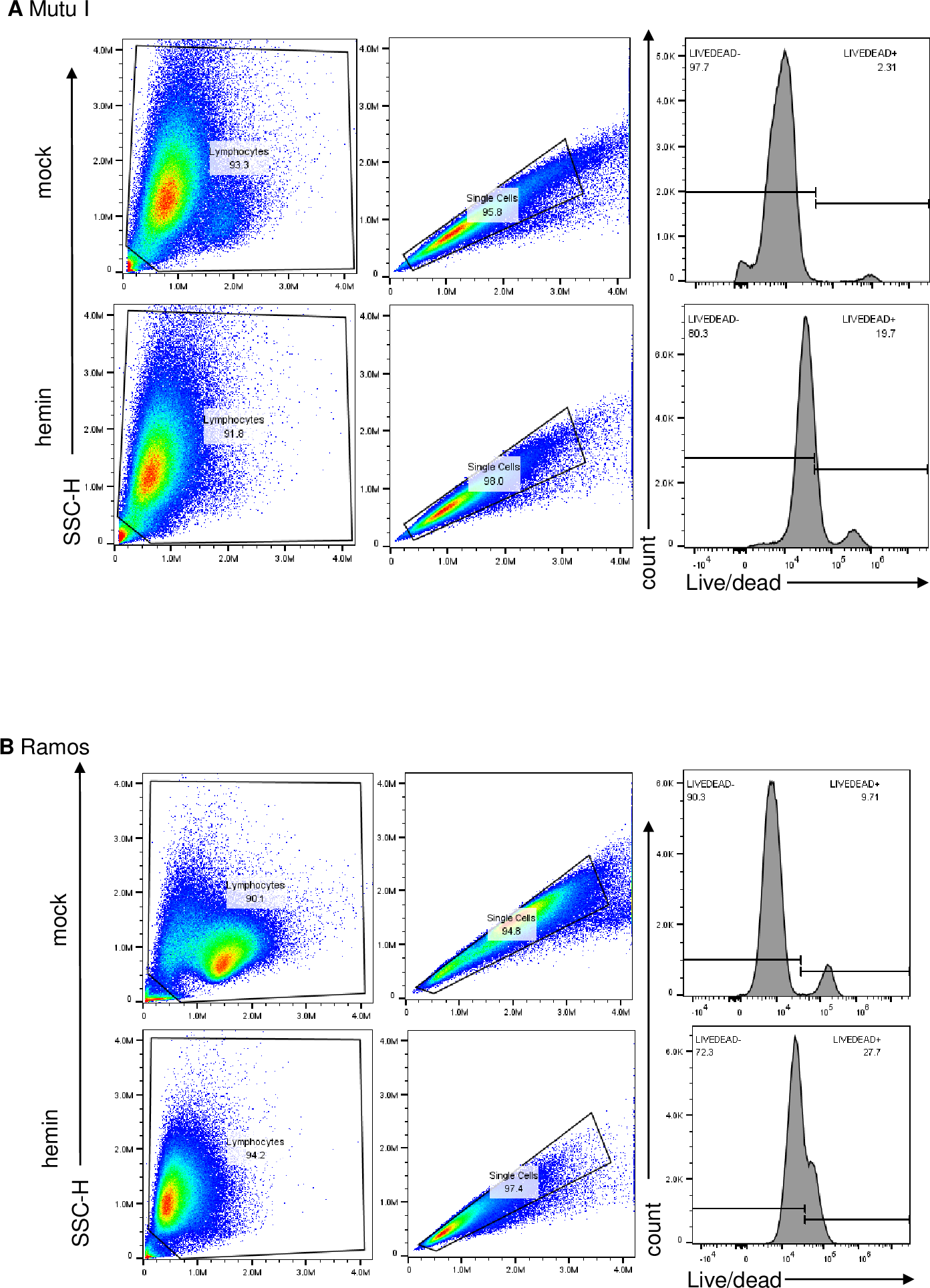

Supplement: S4 Fig — Mutu I (A) and Ramos (B) BL cell lines were analyzed in FlowJo by gating in the order of lymphocytes, single cells, then live dead. (TIF) [file ppat.1011561.s004.tif]

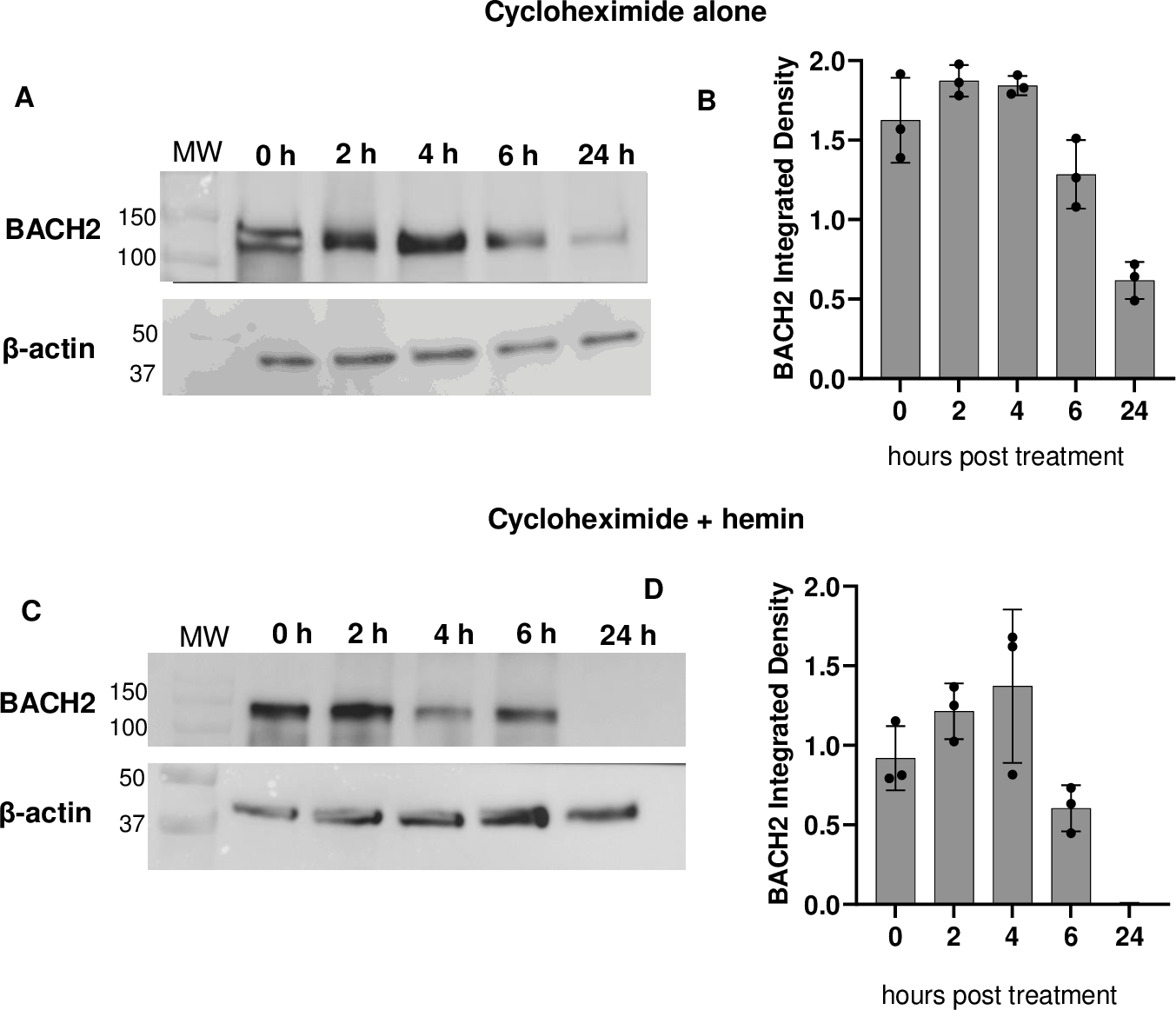

Supplement: S5 Fig — Stability of BACH2 was measured using cycloheximide (CHX) alone (A) and quantified (B) and in combination with hemin (C) and quantified (D). N = 3 for all western blots. (TIF) [file ppat.1011561.s005.tif]

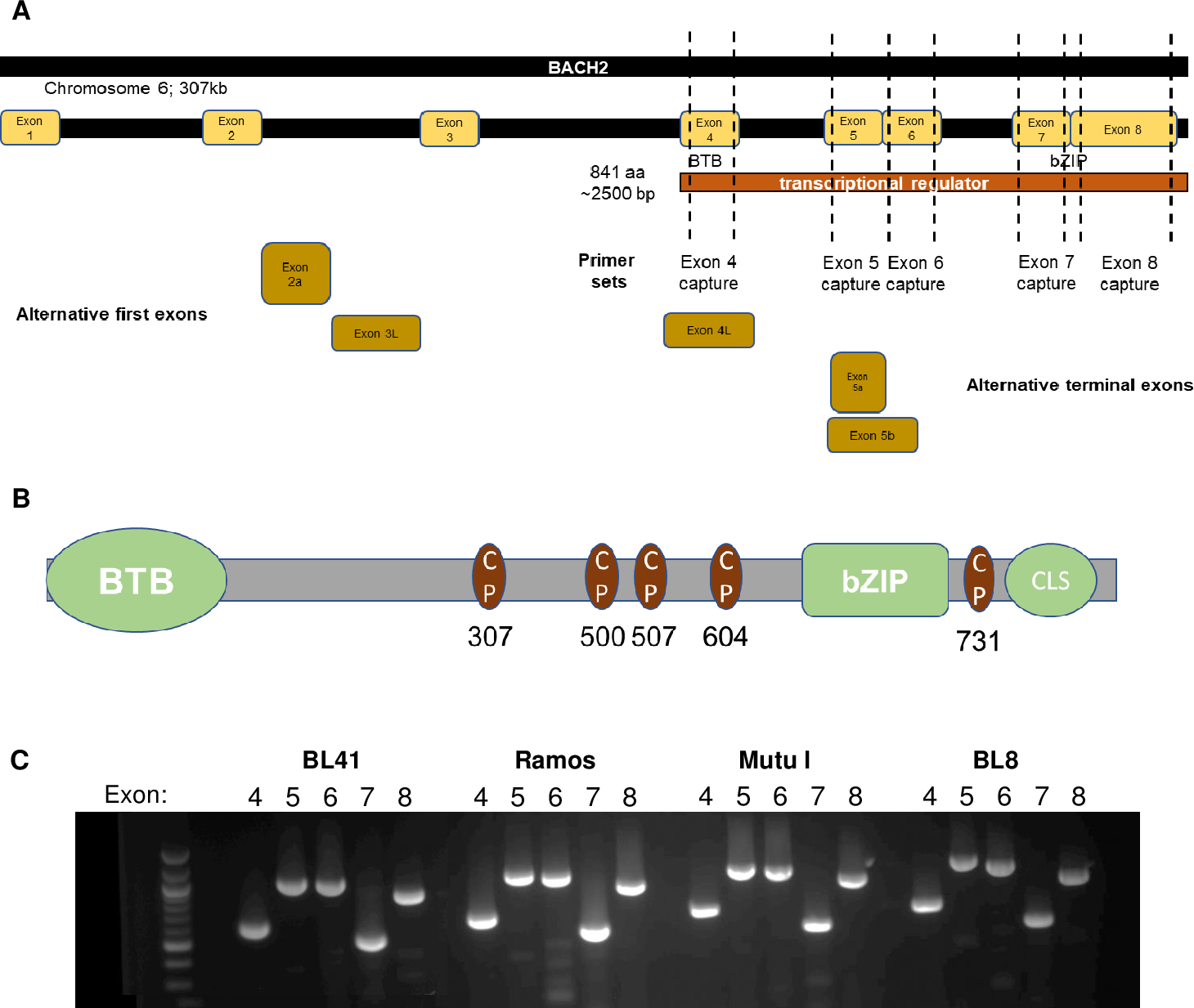

Supplement: S6 Fig — (A) Schematic of the BACH2 gene, corresponding mRNA with exons shown in yellow, and the resulting transcriptional regulator protein shown in tan. Dotted lines represent primers used to capture exons that encode for the transcriptional regulator. (B) Schematic of the BACH2 transcriptional regulator with prominent features in green (BTB domain, bZIP- basic leucine zipper, CLS-cytoplasmic localization signal) and the five cysteine-proline motifs that serve as heme binding sites shown in red, labeled with their amino acid positions. (C) PCR products from exons 4 through 8 for each cell line were run on a DNA agarose gel. (TIF) [file ppat.1011561.s006.tif]

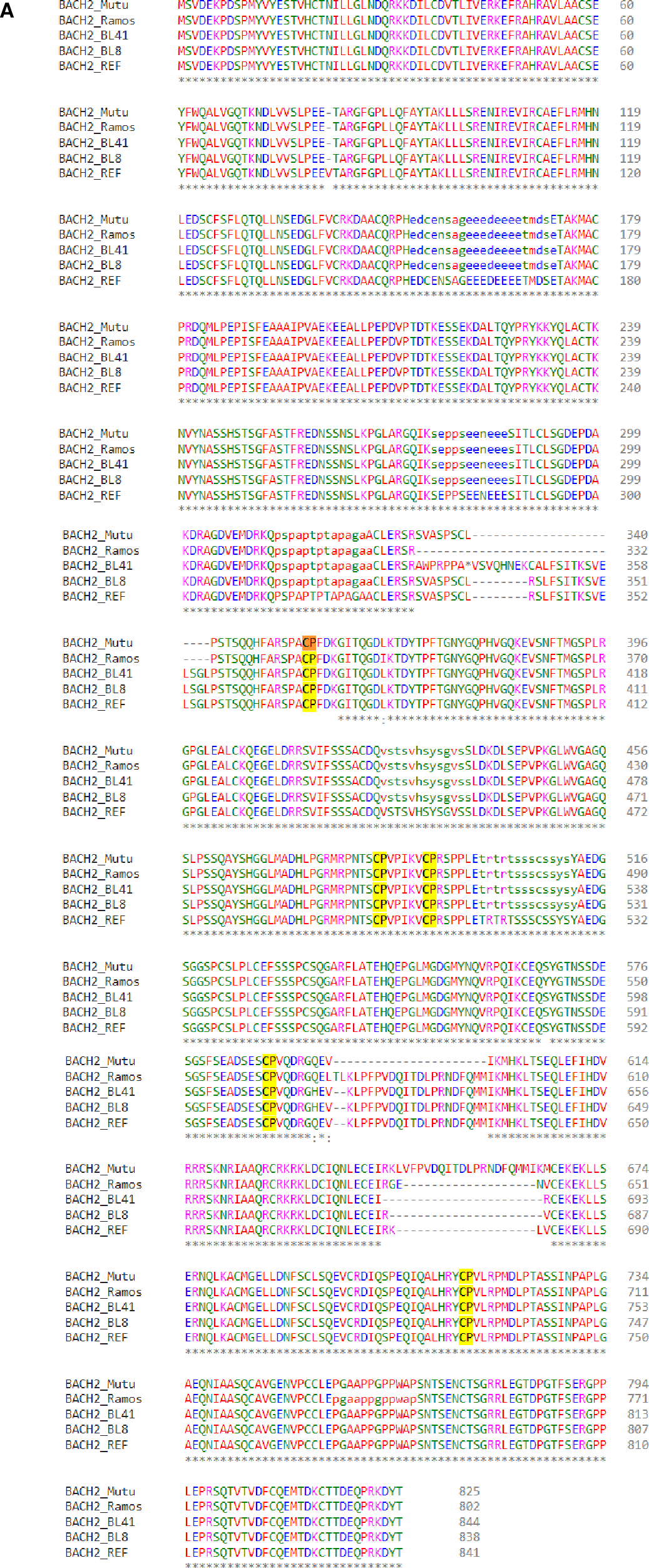

Supplement: S7 Fig — BACH2 transcriptional regulator sequences derived from Mutu I, Ramos, BL41, and BL8 and compared to the NCBI BACH2 reference sequence. Cysteine proline motifs that serve as heme binding sites are highlighted in yellow. (TIF) [file ppat.1011561.s007.tif]

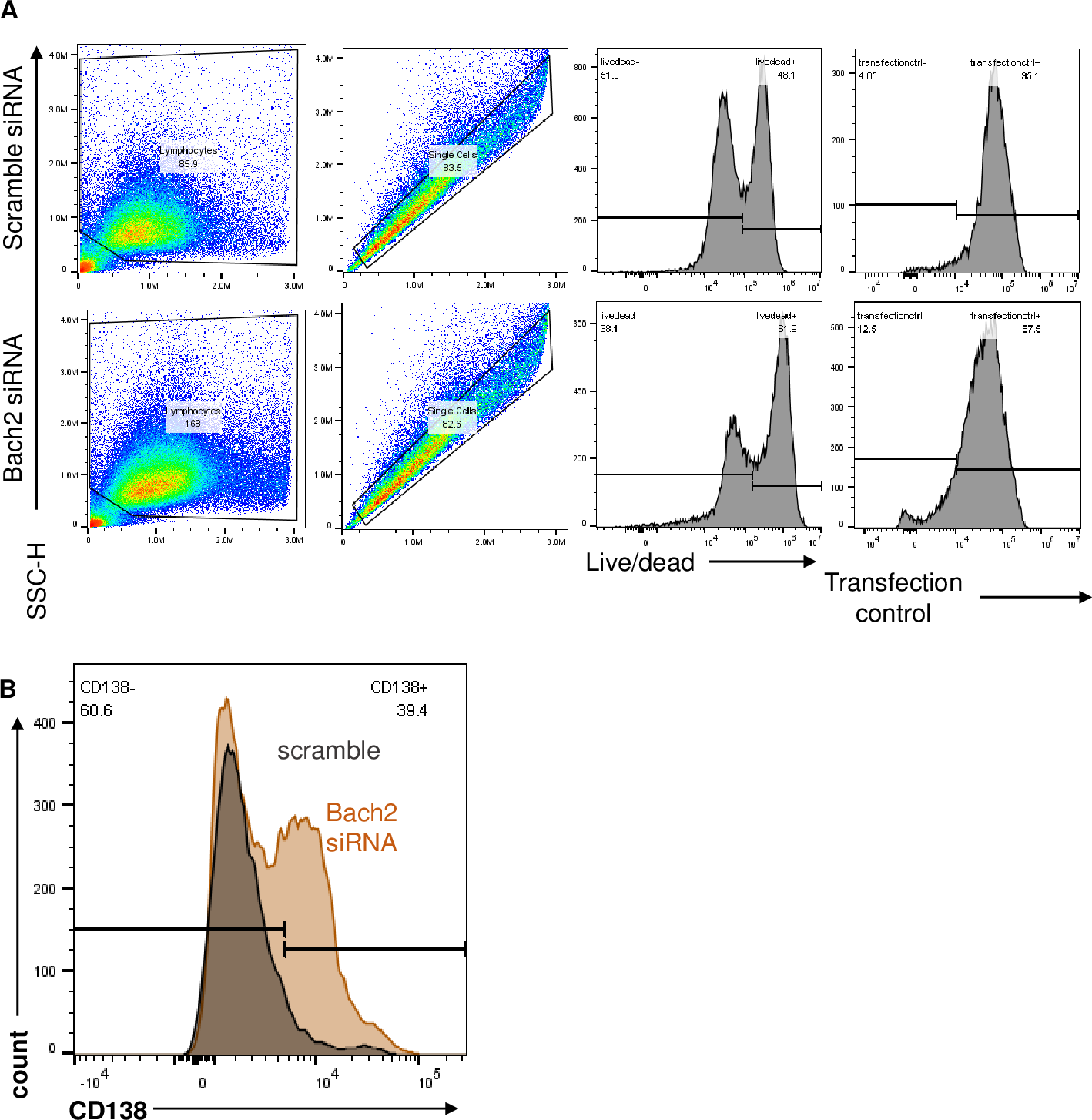

Supplement: S8 Fig — (A) BACH2 siRNA or scramble cells were gated on lymphocytes, single cells, live cells, then transfected cells. (B) CD138 expression compared between scramble and BACH2 siRNA. (TIF) [file ppat.1011561.s008.tif]
